# Chemistry-wide association studies (CWAS) to determine joint toxicity effects of co-occurring chemical features

**DOI:** 10.1186/1758-2946-6-S1-P15

**Published:** 2014-03-11

**Authors:** Yen Low, Alexander Sedykh, Denis Fourches, Alexander Tropsha

**Affiliations:** 1 Laboratory for Molecular Modeling, Division of Chemical Biology and Medicinal Chemistry, UNC Eshelman School of Pharmacy, University of North Carolina at Chapel Hill, Chapel Hill, NC, 27599, USA

## 

Individual structural alerts often fail to accurately predict chemical toxicity as they tend to overlook the moderating effects of other co-occurring alerts. Features are said to have statistical interaction effects when one changes or modulates the effect of another on the target property. Here we introduce Chemistry-Wide Association Study (CWAS; by analogy with GWAS in genomics) to systematically elicit the individual and interaction effects of chemical features on the target property. A mutagenicity dataset of 5,439 compounds was used in this proof-of-concept study. We utilized QSAR models built with random forest and ISIDA fragment descriptors to select the most important chemical features and identify pairs of features with significant interaction effects. These interacting feature pairs revealed how subtle structural changes affect mutagenicity (e.g., ortho-substitution reduces mutagenicity caused by nitroarene moiety). We also found that feature pairs can be integrated into more specific structural alerts with fewer false positives. We believe the interaction effects uncovered by CWAS are useful for refining structural alerts and enhancing model interpretation, enabling more effective design of safe chemical substances and leading to the improved regulatory chemical risk assessment.

